# Factors Related to Seeking Help for Postpartum Depression: A Secondary Analysis of New York City PRAMS Data

**DOI:** 10.3390/ijerph17249328

**Published:** 2020-12-13

**Authors:** Silvia Manso-Córdoba, Sarah Pickering, Miguel A. Ortega, Ángel Asúnsolo, Diana Romero

**Affiliations:** 1 Department of Surgery, Medical and Social Sciences, Faculty of Medicine and Health Sciences, University of Alcalá, Alcalá de Henares, 28801 Madrid, Spain; s.manso96@gmail.com; 2 Graduate School of Public Health and Health Policy, City University of New York (CUNY), New York, NY 10010, USA; sarah.pickering34@sphmail.cuny.edu; 3 Department of Medicine and Medical Specialties, Faculty of Medicine and Health Sciences, University of Alcalá, Alcalá de Henares, 28801 Madrid, Spain; miguel.angel.ortega92@gmail.com; 4 Ramón y Cajal Institute of Healthcare Research (IRYCIS), 28034 Madrid, Spain; 5 University Center for the Defense of Madrid (CUD-ACD), 28047 Madrid, Spain; 6 Department of Community Health and Social Sciences, City University of New York (CUNY) Graduate School of Public Health and Health Policy, New York, NY 10010, USA

**Keywords:** postpartum depression, stigma, women’s health, pregnancy, mental health

## Abstract

Postpartum depression (PPD) affects 13% of mothers and can have a major impact on their lives and those of their children. However, most cases go undiagnosed, and the risk factors for this underdiagnosis are not yet fully known. We intended to analyze the influence of different sociodemographic and health factors associated with symptoms of postpartum depression. Data from the New York City Pregnancy Risk Assessment Monitoring System (PRAMS) for 2016–2017 were analyzed. 618 women met the inclusion criterion of recurring depressive symptoms. Most women who experienced PPD symptoms did not seek help. Seeking help was a much better predictor of the diagnosis of PPD when compared to questions regarding symptoms. The most important factors related to a decreased risk of not asking for help were having a previous mental health history and having doctor visits for a chronic illness. The racial group most at risk of not asking for help were Asian/Pacific Islander (API) women. Interventions aimed at reducing the stigma and increasing knowledge of PPD should be incorporated into the antenatal education of expectant mothers, particularly among women who may not have previously sought care for mental or chronic illnesses.

## 1. Introduction

Childbirth and the puerperium are periods of extraordinary neuroendocrine and psychosocial changes for women, which predispose them to the appearance of various conditions. Of these, postpartum depression (PPD) is perhaps one of the most tragic, both in terms of its prevalence and the impact it can have. The World Health Organization reports around 13% of women who have given birth suffer from it, with the prevalence closer to 20% in developing countries [1,2].

It is essential that we do not mistake postpartum depression for the so-called postpartum blues or maternity blues, which occurs in 70–80% of postpartum women a few days after childbirth and disappears spontaneously without causing functional impairment or aftereffects. We must also distinguish it from another even more serious puerperal pathology, postpartum psychosis, which occurs in 1 out of every 1000 deliveries [3].

PPD begins weeks or months after childbirth. The duration is usually limited, with symptoms including feelings of sadness, guilt, constant worry, anxiety, and insomnia. While most cases are diagnosed as major depression with concomitant generalized anxiety, one fifth of PPDs have an underlying bipolar disorder [4]. In some cases of postpartum depression, there is even a risk to the mother’s life, with up to 5% of patients reporting frequent autolytic ideation [5]. In fact, suicide is one of the main causes of death during this period [6,7].

Maternal depression has been linked to feelings of inadequacy for child care and ambivalence towards the child. The mother–child relationship can take a toll, and this can lead to problems in the child’s development: delayed acquisition of skills, academic problems, socialization difficulties, behavioral disturbances, and substance abuse [8,9,10].

The etiopathogenesis of PPD has been attributed to the hormonal alterations associated with the postpartum period. The most promising evidence points to the deficit of a progesterone derivative, allopregnanolone, known to stimulate the α4 subunit of the GABAA receptor, giving it antidepressant, anxiolytic, sedative, and neuroprotective effects [11,12]. In 2019, the United States (US) FDA approved the intravenous infusion of allopregnanolone, also called brexanolone, for the treatment of postpartum depression [13,14,15].

Despite the numerous developments in the management of PPD that have taken place in recent years, it is still a deeply underdiagnosed condition. In 2014, a US survey answered by 1400 women revealed that 40% of those with symptoms of depression did not seek help. Their reasons included shame, guilt, not caring, not thinking it required treatment, and fear of being stigmatized for having a mental illness [16]. In 2005, a Swedish narrative study of 22 mothers had observed that they did not consult health services for fear of being perceived as weak or bad mothers [17]. These findings provide support for a lack of awareness and stigma as contributors to the under-reporting of PPD.

The stigmatization of mental health conditions has been identified as a barrier to diagnosis and treatment [18]. Negative prejudices about these patients and the idea that they are responsible for their illness prevent many people from recognizing their own symptoms and seeking help [19]. However, women who experience postnatal depressive symptoms seem to show more openness toward acknowledging these kinds of health issues [20]. Moreover, a history of pre-pregnancy depression has been associated with a greater likelihood of diagnosis in women with PPD [21].

Some authors have suggested that individuals’ contact with mental healthcare services and the receipt of related information are associated with positive changes in attitude. Indeed, mental health literature is linked to a reduction in mental health stigma [22], as well as an increase in help-seeking attitudes, both professionally and amongst family and friends [23].

Of further concern are the racial and ethnic disparities in PPD. Although differences in the prevalence of PPD among ethnic and racial groups vary across studies [24,25], there seems to be some consensus about differences in its management. However, even among racial/ethnic groups with similar economic and educational levels, white mothers enjoy higher rates of care and follow-up for their PPD compared to their black and Hispanic counterparts [26].

It is possible to attribute part of these differences to cultural factors related to mental health; when faced with this type of issue, Hispanic people tend to rely more frequently on their family for assistance, and it is not uncommon for Asian communities to avoid talking openly about these issues [27,28]. However, African Americans show positive attitudes towards seeking help from medical professionals [27,29,30], which contrasts with their under-utilization of mental health services [31]. In the case of PPD, it has been argued that the fear of seeking help arises from the notion that large numbers of African American children end up in foster care programs [32,33].

While the prevalence of PPD in different population groups is a frequent topic of study, the risk factors for its underdiagnosis, as well as possible interventions to achieve greater detection of it, have not yet been studied in depth. For this reason, the first aim of our research was to determine which subgroups of women are most at risk of not seeking professional help when they experience symptoms compatible with PPD. We hypothesized that women from racial and ethnic minority backgrounds would be more at risk for not asking for help for PPD than white women. Additionally, a second aim of this research was to investigate the possible impact of health professionals asking mothers about PPD symptoms. In this regard, we hypothesized that women who were asked about symptoms of PPD by healthcare providers would be more likely to seek mental health services than those who were not asked.

## 2. Materials and Methods

### 2.1. Study Design and Sample

This study was a secondary analysis of data obtained from PRAMS (Pregnancy Risk Assessment Monitoring System), a periodic population surveillance system of the CDC (US Centers for Disease Control and Prevention) that collects information from women who have given birth to live newborns in 47 US states and New York City (NYC) [34]. For our study, we used data from Phase 8 of PRAMS (2016–2017) in NYC.

To collect this data, a self-administered survey is mailed on a monthly basis to a random sample of women in NYC within 2–4 months of giving birth. For those women who do not respond to the written questionnaire, a telephone interview is conducted. Respondents are given an incentive for their participation; a response rate over 70% was achieved. PRAMS data are weighted to be representative of women who gave birth in NYC during the study year. The survey includes questions about socio-demographic factors and data concerning their health, as well as attitudes and experiences before, during, and after pregnancy, which are associated with information obtained from birth certificates.

For the purposes of this study, the analytic sample comprised women who reported experiencing postpartum depressive symptoms (PDS). Specifically, this pertained to women who answered “occasionally,” “often/ almost always,” or “always” to the question: “Since your baby was born, how often have you felt depressed, sad, or hopeless?”

### 2.2. Measures

The dependent variable of seeking care for depression was the question: “Since your baby was born, have you sought help for your depression from a doctor, nurse, or other health professional?” We were specifically interested in factors associated with not having asked for help for depression.

Socio-demographic variables included age (<20, 20–35, >35), years of education (<8, 8–15, >15), annual family income (<$20,000, $20,001–60,000, >60,000), race/ethnicity (white, black, Hispanic, Asian/Pacific Islander (API), other), Medicaid (a US health insurance program for people with limited resources), and WIC (a US nutritional assistance program for mothers and young children below the poverty line).

Pregnancy-related variables included the intention to become pregnant (“When you became pregnant with your new baby, were you looking to become pregnant?”) and parity based on the number of previous children (first pregnancy if none; multiparous if ≥1).

Health-related factors that were taken into account included having made visits in the previous year for depression and/or anxiety, chronic illnesses, check-ups with a general practitioner, gynecological check-ups, and/or a postpartum check-up, perception of one’s own health (excellent, very good, good, regular, bad), diagnosis of pre-pregnancy depression and/or anxiety, and diagnosis of postpartum depression (“Since your new baby was born, has a doctor, nurse, or other healthcare professional told you that you have depression?”). We also examined whether they were asked about symptoms of depression during pregnancy (“During any of your prenatal care visits, did any doctor, nurse, or other health professional ask you if you were depressed or sad?”) and after delivery (“During your postpartum checkup, did any doctor, nurse, or other health professional ask you if you were depressed or sad?”).

### 2.3. Statistical Analysis

We carried out a descriptive analysis of the sample, taking into account the absolute and relative frequencies of the different variables, which were compared using the chi-square or Fisher’s exact test, as appropriate. We considered a value of *p* < 0.05 as statistically significant and used 95% confidence intervals (CIs) to assess the statistical significance of the calculated odds ratios. Data analysis was performed using SPSS Statistics V. 26 (IBM Corp, Armonk, NY, USA) software.

## 3. Results

### 3.1. Sample Size and Prevalence

Among the 2729 women who completed the NYC Phase 8 PRAMS survey, 618 (22.6%) met the inclusion criterion for our study, i.e., recurrent feelings of sadness since the birth of their baby. Of these 618 mothers with symptoms, a total of 89 women (14.4%) had been diagnosed with depression after childbirth and 112 (18.1%) had sought help from a healthcare professional. We also found that most of these women had been asked about symptoms of depression: 390 mothers (63.1%) stated that they had been asked during a prenatal visit and 375 (60.7%) at the postpartum check-up.

Table 1 shows the association between the diagnosis of PPD and having been asked during pregnancy, having been asked after giving birth, and having asked for help. Although all factors appear to significantly increase the probability of diagnosis, asking for help led to the highest rates of diagnosis (OR = 19.492, CI = 11.448, 33.187, *p* < 0.001) compared to being asked during an antenatal visit (OR = 1.684, CI = 0.995, 2.848, *p* < 0.05) or at the postpartum check-up (OR = 2.565, CI = 1.372, 4.795, *p* < 0.05).

### 3.2. Depending on the Frequency of the Symptoms

As one might expect, there was a positive association between the highest recurrence of symptoms and the request for help (*p* < 0.001). Of the 112 women who did ask for help, the group with the highest rate of request were those reporting symptoms “almost always” (38 of 82 (6.3%)), followed by the group experiencing symptoms “always” (11 of 25 (44.0%)), and finally, those with symptoms “occasionally” (63 of 506 (12.5%)). In all three groups, the request for help was positively associated with the diagnosis of postpartum depression: “occasionally” with OR = 21.465 (CI = 10.838, 42.509, *p* < 0.01), “almost always” with OR = 5.963 (CI = 2.239, 15.882, *p* < 0.01), and “always” with OR = 22.750 (CI = 2.114, 244.868, *p* < 0.05).

### 3.3. Sociodemographic Variables

The demographic characteristics of the analytic sample can be seen in Supplementary Materials Tables S1 and S2. The variable most strongly associated with seeking help for depression was race/ethnicity: 23.0% of white women, 21.8% of Hispanic women, 16.8% of black women, and 8.5% of API women sought help. API women showed a higher risk of not asking for help compared with white women (OR = 3.187, CI = 1.530, 6.640, *p* < 0.001); there was no statistically significant difference among the other racial/ethnic groups (Table 2).

Women with incomes of US$60,000 and higher were significantly less likely to not seek help (OR = 0.482, CI = 0.274, 0.849, *p* < 0.05) compared to their lower income counterparts. In contrast, women with intended pregnancies showed an increased risk of not seeking help for depression (OR = 1.648, CI = 1.112, 2.548, *p* < 0.05). Neither age, education, nor number of previous pregnancies were significantly associated with seeking help for depression.

### 3.4. Health Variables

Supplementary Materials Table S3 contains the health-related traits and experiences of the women in the study sample. As shown in Table 3, depression (OR = 0.291, CI = 0.176, 0.480, *p* < 0.001), anxiety (OR = 0.238, CI = 0.152, 0.371, *p* < 0.001), doctor visits for depression and/or anxiety in the last year (OR = 0.119, CI = 0.060, 0.236, *p* < 0.001), and visits for chronic illnesses (OR = 0.274, CI = 0.139, 0.540, *p* < 0.001) were positively related to asking for help. To rule out the influence of a possible association between chronic disease and mental illness, we subsequently analyzed only women with visits for chronic disease without mental health diagnosis or visits to these services; the visits for chronic illnesses maintained their protective effect after making this adjustment (OR = 0.364, CI = 0.166, 0.798, *p* < 0.01). Self-perception of health status also achieved statistical significance (OR = 0.484, CI = 0.263, 0.889, *p* < 0.05), although this was linked to the presence of mental pathology prior to pregnancy.

On the other hand, general medical visits, gynecological check-ups, and asking for symptoms of depression at any prenatal or postpartum check-up were not associated with a reduction in the risk of not seeking professional help for depression. The same was found for Medicaid health insurance and WIC assistance.

### 3.5. Risk Factors Based on Race and Ethnicity

On the basis of the socio-demographic and health disparities between the various racial/ethnic groups, which can be found in Supplementary Materials Tables S4 and S5, respectively, we carried out a risk analysis stratified by race. For the socio-demographic variables (Table 4), age above 35 in whites was associated with a lower risk of not seeking help (OR = 0.468, CI = 0.233, 0.945, *p* < 0.05). In Hispanic women, an intended pregnancy (OR = 2.312, CI = 1.061, 5.037, *p* < 0.05) and multiparity (OR = 2.059, CI = 0.995, 4.262, *p* < 0.05) were associated with increased risk of not seeking help. Among African Americans, both the lowest (OR = 0.177, CI = 0.037, 0.859, *p* < 0.05) and highest income levels (OR = 0.136, CI = 0.024, 0.778, *p* < 0.05) were associated with reduced risk, i.e., middle-class black women were at risk of not seeking care. Among API women, there were no significant differences for any of these socio-demographic variables.

Next, we analyzed the association between health-related factors and the risk of not seeking help for depression, stratifying by race/ethnicity (Table 5). In the white subgroup, women with poor health perception (OR = 0.188, CI = 0.030, 1.169, *p* < 0.05), history of depression (OR = 0.243, CI = 0.100, 0.588, *p* < 0.01), history of anxiety (OR = 0.190, CI = 0.090, 0.402, *p* < 0.01), had made a medical visit for depression and/or anxiety in the 12 months before pregnancy (OR = 0.144, CI = 0.055, 0.380, *p* < 0.01), or had a visit for chronic disease (OR = 0.330, CI = 0.128, 0.852, *p* < 0.05) were at lower risk of not seeking help. Hispanic mothers showed notable similarities to white mothers, with the variables associated with lower risk of not seeking help being depression (OR = 0.305, CI = 0.136, 0.687, *p* < 0.01), anxiety (OR = 0.294, CI = 0.137, 0.628, *p* < 0.01), depression and/or anxiety visit (OR = 0.103, CI = 0.025, 0.416, *p* < 0.01), and a chronic illness visit (OR = 0.261, CI = 0.185, 0.368, *p* < 0.01).

Within the African American group, however, only those with chronic illness visits (OR = 0.194, CI = 0.036, 0.1064, *p* < 0.05) showed a statistically significant reduction in the risk of not seeking help for depression. Asian (API) women stood out in that Medicaid-type insurance (OR = 7.875, CI = 0.963, 64.389, *p* < 0.05) and having attended a postpartum check-up (OR = 11.111, CI = 2.455, 50.283, *p* < 0.05) were associated with an increased risk of not seeking help. More in line with the other racial/ethnic group findings was that API women’s visits for depression and/or anxiety were associated with a reduced risk of not seeking help (OR = 0.052, CI = 0.020, 0.133, *p* < 0.05).

## 4. Discussion

In order to adequately diagnosis any disease, there must not only be accessible health services, but also demand for these services by those who are sick. This study has found that, in the case of PPD and unlike other diseases, the main factor to improve diagnosis likely resides in increasing the demand for healthcare services among women with symptoms, that is, in reducing the social stigma of mental illness. This sub-sample of 618 postpartum NYC women with recurrent depressive symptoms illustrates the diversity of PPD and the factors linked to its lack of diagnosis. In this sample, only 14% (89 of 618) of the mothers were diagnosed with depression. In fact, when we look at the total survey sample (n = 2729), the rate of diagnosed PPD is 4.7% (n = 128), much lower than the average prevalence for this pathology, which is around 13%. In other words, we are not diagnosing most of the mothers who suffer from it.

As in previous studies [21], asking about symptoms at ante (OR = 1.48) and postnatal care visits (OR = 2.565) was associated with an increased likelihood of detection. However, our data suggest that the most important determinant for the diagnosis of PPD is the woman’s request for help (OR = 19.492). Previous authors have found that mothers who suffer from PPD are less likely to admit their symptoms and ask for help [35]. As we pointed out in the introduction, there is much guilt and shame surrounding this condition, which makes it difficult for many women to talk about it. Borrowing a mother’s quote from research conducted by Edwards et al.: “I was worried about what people thought, that I wasn’t dealing with having a baby, I don’t know why I was so paranoid about it, but I didn’t want people to know I had a mental illness” [20]. In our study, only 18% of those with symptoms sought help.

The question then arises as to what factors influence the likelihood of seeking help for PPD in order to identify interventions that may increase the detection of this condition. At the start of this project, we considered that unintended pregnancy could be associated with an increased risk of not seeking help for depression, given that previous studies had shown that unintended pregnancies were at greater risk for depression [36]. In addition, these types of pregnancies tend to occur more in women with lower economic status [37], which is traditionally considered a risk factor for not seeking medical help for PPD.

Women with unintended pregnancies often find themselves stigmatized by their environment, and may lack the social support that other mothers receive [38]. As earlier research suggests, greater social support has been tied to higher rates of seeking help for PPD [39]. Therefore, it may seem reasonable to assume that unintended pregnancies would have an increased risk of not being diagnosed with PPD. However, this is not what we observed in our study sample. Interestingly, it was women with intended pregnancies who were more likely not to ask for help (OR = 1.683). Even more pronounced is this increased risk for Hispanic women (OR = 2.312).

We posit that it is possible that women who have been seeking to become pregnant have come to generate certain expectations in themselves and in the environment they planned to provide for their newborn. Consequently, they may feel more guilt and shame when they experience negative feelings after the birth of their child. In order to test this hypothesis, future studies are needed to explicitly investigate this phenomenon. Although similar studies have found large racial and ethnic differences in help-seeking rates [40], in our sample, we found similar help-seeking attitudes among white women (23.0%) and Hispanic women (21.8%), and a moderate tendency for black women to ask for less help (16.8%).

The one racial group that stood out from the others was API women, with only 8.5% seeking help for depression. Women in the API subgroup had a higher risk of not asking for help (OR = 3.187) than white women, consistent with results from previous studies that have observed a lower tendency of the Asian population to talk about mental health and ask for help [27,41]. Augsberget et al. identified family and community pressure as the main barriers, but also the lack of more holistic and culturally sensitive therapy [41]. In Spain, the Asian community is growing, with more than a quarter million people living here [42,43]. Our results, which are corroborated with the main findings of the literature, underline the need to incorporate strategies specifically aimed at promoting the use of mental health services by this subgroup.

As we mentioned in the Introduction, a history of mental illness (depression, OR = 0.291; anxiety, OR = 0.238) was positively correlated with seeking help. Unfortunately, statistical significance was not achieved for black women or for API women, probably due to the low prevalence of this type of mental illness history in both subgroups. Medical visits for depression and/or anxiety (OR = 0.119) favored help-seeking in all groups except African-American women. There may be a connection between having overcome the stigma of a mental illness and facing the challenge of PPD.

Our data show an association between asking for help and visits due to chronic illness (OR = 0.364), regardless of their association with mental pathologies. The presence of stigma has already been reported in many chronic illnesses, particularly in neurological [43,44], autoimmune [45], and HIV [46]. People living with these diseases report a negative impact on their lives beyond the organic symptoms: distancing from family and friends, worsening of relationships at work, and lower self-esteem [47,48].

This surprising finding may suggest that overcoming the barrier of “being sick” might help mothers overcome the fear of asking for help for their PPD. We believe that there is a need for strategies that will improve visibility and knowledge about PPD. The data from our study have shown that it is not enough to ask about these symptoms. If we want to help these women, our best intervention option would be to eliminate the stigma associated with PPD, which would not only increase the likelihood of diagnosis, but also subsequent adherence to treatment [48,49].

Two possible routes have been suggested to achieve these goals: mental health education of mothers in prenatal classes [35] and interviews conducted during pediatric visits [50]. The American Academy of Pediatrics recommends that mothers be screened for PPD when they come for check-ups during the first six months after childbirth [51]. Although most women seem to support pediatricians doing the screening, they have some suggestions on how to do it. They recommend that these doctors receive specific training on the management of postpartum depression, and that they establish a good doctor–patient relationship to carry out this screening, instead of conducting a “cookie-cutter” test [50].

We believe that obstetric visits and prenatal education classes are excellent platforms for providing information on PPD. Just as we inform these women about other conditions and symptoms they should be able to recognize, we also need to discuss the possibility of developing depression during pregnancy or after delivery, and aim to normalize it as much as possible. It is important to establish a relationship of trust so that they feel comfortable in communicating any symptoms or discomfort they may feel. To get a more accurate understanding of the reasons why mothers with PPD do not seek help or treat their depression, we need additional studies that capture the views of these women.

Although this research expands our knowledge of PPD, it is not without its limitations. First, the self-administered nature of the survey may contain inaccuracies. For instance, it would have been preferable to corroborate the clinical data with the participants’ medical records. While participant responses could be subject to recall bias, a three-year calendar is included with the questionnaire as a memory aid. In addition, questionnaires completed nine or more months after delivery are not accepted to minimize the potential for recall bias. Likewise, the PRAMS study is explicitly designed to reduce the likelihood of recruitment bias by conducting up to three rounds of mailed questionnaires, followed by several calls to potential participants and possible completion by telephone in addition to mail. This allows for successful recruitment of a broader range of participants and, therefore, a less biased sample.

Another limitation is that the sample focuses on women in NYC, which entails a particular cultural context that may not be extrapolated to other populations, such as those in more rural areas. Additionally, PRAMS excludes mothers who have given birth to babies who died during pregnancy or delivery, so their possible experiences with PPD are not represented in our analysis.

Additionally, our sample only includes women who have felt depressed, hopeless, or sad, without including other common symptoms of postpartum depression, such as anxiety. The most appropriate approach would have been for PRAMS to have used the tool traditionally used in PPD screening, the Edinburgh Postnatal Depression Scale (EPDS), which includes 10 items based on the most common manifestations of PPD, such as sadness, guilt, sleep disturbance, and even self-harm ideation. Indeed, the prevalence of PPD in our sample (22.6%) was higher than that estimated for the general population, probably due to the use of a one-dimensional criterion rather than a combination of factors representing the complex nature of PPD [52]. Furthermore, we have restricted the study of these types of symptoms in the postpartum period, while the DSM-V includes this condition within peripartum depression, which includes women who suffer from depression during pregnancy or in the first two months after childbirth [53].

## 5. Conclusions

Postpartum depression is not only a frequent and potentially serious pathology for both mother and child, but it is also highly under-diagnosed. The guilt and shame associated with PPD seem to be the greatest barriers these women face in seeking help.

Our findings highlight the need to increase awareness among pregnant women of the importance of PPD so that they can seek help, as asking about these symptoms by clinicians is not enough. We also found that there are particularly vulnerable sub-populations, such as API mothers, those with intended pregnancies, or women with no previous contact with mental health services, who are at an increased risk of not seeking care.

All would agree that population health is influenced by many factors, with the accessibility and quality of health services among them. Our results support the need for awareness raising and educational interventions as an essential element for improving access to quality mental healthcare to women in the prenatal and postpartum periods. This will require interventions aimed at reducing the social stigma of depression through community-based campaigns, which take into account racial/ethnic, socioeconomic, and other cultural differences across groups that emerged in this study. While this study is only one piece of the puzzle, it provides a framework for future studies investigating the mechanisms underlying the under-diagnosis of PPD, as well as how to counteract the stigma and lack of treatment of this condition. Our clinical and public health efforts should now focus on raising awareness of PPD among all expectant mothers and offering them our support as health professionals.

## Figures and Tables

**Table 1:** Diagnosis of depression in women with recurrent symptoms as reported in NYC PRAMS (Phase 8).

	OR	95% Confidence Interval		*p*-Value
		Lower	Upper	
Being asked at the prenatal visit	1.68	0.99	2.85	<0.05
Being asked at post-natal check-up	2.56	1.37	4.79	<0.05
Seeking help	19.49	11.44	33.19	<0.001

**Table 2:** Association between not asking for help for depression and socio-demographic characteristics.

	OR	95% Confidence Interval		*p*-Value
		Lower	Upper	
Age (years)				
20–35	1			
<20	0.746	0.203	2.744	0.658
>35	0.770	0.503	1.180	0.229
Education (years)				
8–15	1			
<8	0.444	0.193	1.024	0.052
>15	0.674	0.427	1.064	0.089
Household income (US$)				
20.001–60.000	1			
<20.000	0.581	0.318	1.061	0.075
>60.001	0.482	0.274	0.849	< 0.05
Pregnancy status				
Unintended	1			
Intended	1.683	1.112	2.548	< 0.05
Parity				
First pregnancy	1			
Multiparous	1.191	0.790	1.750	0.403
Race/ethnicity				
White	1			
Hispanic	1.066	0.648	1.754	0.801
Black	1.476	0.832	2.618	0.182
API	3.187	1.530	6.640	<0.001

**Table 3:** Association between not asking for help for depression and health-related characteristics.

	OR	95% Confidence Interval		*p*-Value
		Lower	Upper	
Poor perception of health	0.484	0.263	0.889	<0.05
Poor perception of health after adjustment for mental health factors	0.436	0.132	1.438	0.162
Postpartum Checkup	1.105	0.580	2.105	0.761
Depression (pre-pregnancy)	0.291	0.176	0.480	<0.001
Anxiety (pre-pregnancy)	0.238	0.152	0.371	<0.001
Visit for depression/anxiety	0.119	0.060	0.236	<0.001
Visit for chronic illness	0.274	0.139	0.540	<0.001
Visit for chronic illness after adjusting for mental health factors	0.364	0.166	0.798	<0.01
Visit for medical check-up	0.785	0.484	1.275	0.328
Visit for gynecological examination	0.895	0.531	1.510	0.678
Being asked about depression prenatally	0.692	0.422	1.135	0.143
Being asked about depression post-partum	0.904	0.579	1.410	0.656

**Table 4:** Association between not asking for help and socio-demographic characteristics stratified by race.

	White			Hispanic			Black			API		
	OR	CI 95%		OR	CI 95%		OR	CI 95%		OR	CI 95%	
		Lower	Upper		Lower	Upper		Lower	Upper		Lower	Upper
Age												
Young adult	1			1			1			1		
Teen	0.821	0.754	0.893	0.447	0.100	1.993	0.795	0.713	0.887	0.930	0.878	0.986
Adult	0.469	0.233	0.945	0.871	0.400	1.897	2.215	0.761	6.449	0.506	0.133	1.930
Education												
8–15 years	1			1								
<8 years	0.260	0.163	0.415	0.379	0.134	1.070	0.789	0.082	7.567	0.962	0.912	1.015
>15 years	1.345	0.547	3.304	0.472	0.217	1.028	0.881	0.351	2.208	0.229	0.028	1.876
Salary												
20.001–60.000 $	1			1			1			1		
<20.000 $	0.944	0.232	3.842	0.643	0.266	1.556	0.177	0.037	0.859	1.185	0.156	8.986
>60.001 $	0.602	0.227	1.598	0.727	0.254	2.082	0.136	0.024	0.778	0.607	0.110	3.359
Intentional	1.697	0.798	3.607	2.312	1.061	5.037	1.774	0.605	5.200	1.207	0.320	4.552
Multiparous	0.855	0.429	1.704	2.059	0.995	4.262	1.422	0.568	3.564	0.932	0.248	3.502

**Table 5:** Association between not asking for help and health characteristics by race.

	White			Hispanic			Black			API		
	OR	CI 95%		OR	CI 95%		OR	CI 95%		OR	CI 95%	
		Lower	Upper		Lower	Upper		Lower	Upper		Lower	Upper
Good health perception	1											
Poor health perception	0.188	0.030	1.168	0.483	0.203	1.145	0.570	0.141	2.301	0.827	0.094	7.281
Medicaid	1.723	0.661	4.490	0.852	0.399	1.820	1.038	0.406	2.655	2.828	0.571	14.009
WIC	2.578	0.734	9.057	0.846	0.398	1.801	0.744	0.280	1.977	7.875	0.963	64.389
Postpartum Checkup	0.659	0.075	5.798	0.544	0.176	1.678	1.278	0.382	4.270	11.111	2.455	50.283
Depression	0.243	0.100	0.588	0.305	0.136	0.687	0.581	0.168	2.005	0.200	0.033	1.199
Anxiety	0.190	0.090	0.402	0.294	0.137	0.628	0.434	0.136	1.390	0.273	0.062	1.211
Visit for depression/anxiety	0.144	0.055	0.380	0.103	0.025	0.416	0.914	0.096	8.741	0.051	0.020	0.133
Visit for chronic illness	0.330	0.128	0.852	0.261	0.185	0.368	0.194	0.036	1.064	0.917	0.855	0.983
Being asked prenatally	0.888	0.444	1.775	1.143	0.467	2.799	0.434	0.093	2.019	1.410	0.374	5.312
Being asked after birth	0.818	0.397	1.686	0.510	0.163	1.595	0.650	0.135	3.126	2.000	0.350	11.418

## Supplementary Materials

The following are available online at https://www.mdpi.com/1660-4601/17/24/9328/s1, Table S1: Prevalence of sociodemographic characteristics in our population, Table S2: Prevalence of health characteristics in our population, Table S3: Prevalence of sociodemographic characteristics based on race, Table S4: Prevalence of health characteristics based on race.

## Abbreviaions

PPD	Postpartum Depression
API	Asian/Pacific Islander
PRAMS	Pregnancy Risk Assessment Monitoring System
CDC	Centers for Disease Control and Prevention
FDA	Food and Drug Administration
WIC	Special Supplemental Nutrition Program for Women, Infants, and Children
OR	Odds Ratio
CI	Confidence Interval
