# Supplementary material for: Factors Related to Seeking Help for Postpartum Depression: A Secondary Analysis of New York City PRAMS Data
Source: Int J Environ Res Public Health. 2020 Dec 13;17(24):9328. doi: 10.3390/ijerph17249328 (PMC7763494; doi:10.3390/ijerph17249328)
Supplement: Supplementary file 1 [file ijerph-17-09328-s001.pdf]

Suppl. Table S1. Prevalence of sociodemographic characteristics in our population.

	Help	No help	p
**Age**			
Teen (<20 years)	3 (2.6%)	11 (21,8%)	0.485
Young adult	70 (62.5%)	344 (68,3%)	
Older adult (>35 years)	39 (34.8%)	148 (29.4%)	
**Education**			
< 8 years	9 (8.0%)	22 (4.3%)	0.241
8-15 years	48 (42.8%)	264 (52.4%)	
>15 years	55 (49.1%)	217 (43.1%)	
**Salary**			
< $20.000	33 (32.3%)	137 (30.5%)	<0.05
$20.001-60.000	20 (19.6%)	143 (31.8%)	
>$60.001	49 (48.0%)	169 (37.6%)	
**Unintended pregnancy**	62 (55.9%)	212 (42.9%)	<0.05
Intentional pregnancy	49 (44.1%)	282 (57.1%)	
**First pregnancy**	59 (52.7%)	243 (48.3%)	0.403
Multiparous	53 (47.3%)	260 (51.7%)	
**Race**			
White	42 (37.5%)	141 (28.0%)	<0.01
Hispanic	38 (33.9%)	136 (27.0%)	
Black	22 (19.6%)	109 (21.7%)	
API	10 (8.9%)	107 (21.3%)	
Others	0 (0.0%)	10 (2.0%)	

Suppl. Table S2. Prevalence of health characteristics in our population.

	Help	No Help	p
Good perception of health	95 (84.8%)	462 (92.0%)	<0.05
**Poor perception of health**	17 (15.1%)	40 (7.9%)	
No Medicaid	69 (63.3%)	271 (58.2%)	0.325
**Medicaid**	40 (36.7%)	195 (41.8%)	
No WIC	68 (60.7%)	271 (54.5%)	0.234
**WIC**	44 (39.3%)	226 (45.5%)	
No postpartum review	13 (11.6%)	53 (10.6%)	0.761
**Postpartum checkup**	99 (88.4%)	446 (89.4%)	
No depression	77 (70.6%)	439 (89.2%)	<0.001
**Depression**	32 (29.3%)	53(10.7%)	
No anxiety	62 (55.8%)	415 (84.1%)	<0.001
**Anxiety**	49 (44.1%)	78 (15.8%)	
No visit for depression / anxiety	66 (71.7%)	321 (95.5%)	<0.001
**Visit for depression / anxiety**	26 (28.2%)	15 (4.4%)	
No visit for disease	74 (80.4%)	315 (93.7%)	<0.001
**Visit for disease**	18 (19.5%)	21 (6.2%)	
No review with a general practitioner	31 (33.7%)	182 (39.3%)	0.328
**Review with a general practitioner**	61 (66.3%)	204 (60.7%)	
No gynecological examination	24 (26.1%)	95 (28.3%)	0.679
**Gynecological examination**	68 (73.9%)	241 (71.7%)	
No prenatal question	35 (32.4%)	166 (34.6%)	0.667
**Prenatal question**	73 (67.6%)	314 (65.4%)	
No postnatal question	26 (26.0%)	146 (32.3%)	0.218
**Postnatal question**	74 (74.0%)	306 (67.7%)	

Supll. Table S3. Prevalence of sociodemographic characteristics based on race.

	White		Hispanic		Black		API	
	Help	No help	Help	No help	Help	No help	Help	No help
**Age**								
Teen	0 (0.0%)	1 (0.7%)	3 (7.9%)	5 (3.7%)	0 (0.0%)	4 (3.7%)	0 (0.0%)	1 (0.9%)
Young adult	21 (50.0%)	96 (68.1%)	26 (68.4%)	97 (71.3%)	17 (77.3%)	66 (60.6%)	6 (60.0%)	80 (74.8%)
Older adult	21 (50.0%)	44 (31.2%)	9 (23.7%)	34 (25.0%)	5 (22.7%)	39 (35.8%)	4 (40.0%)	26 (24.3%)
**Education**								
< 8 years	1 (2.4%)	0 (0.0%)	7 (18.4%)	14 (10.3%)	1 (4.5%)	4 (3.7%)	0 (0.0%)	4 (3.7%)
8-15 years	13 (31.0%)	37 (26.2%)	18 (47.4%)	95 (69.9%)	15 (68.2%)	76 (69.7%)	2 (20%)	51 (47.7%)
>15 years	28 (66.7%)	104 (73.8%)	13 (34.2%)	27 (19.9%)	6 (27.3%)	29 (26.6%)	8 (80.0%)	52 (48.6%)
**Salary**								
< $20.000	4 (9.8%)	17 (13.3%)	17 (48.6%)	47 (40.9%)	10 (58.8%)	39 (39.8%)	2 (22.2%)	32 (32.0%)
$20.001-60.000	6 (14.6%)	27 (21.1%)	10 (28.6%)	43 (37.4%)	2 (11.8%)	44 (44.9%)	2 (22.2%)	27 (27.0%)
>$60.001	31 (75.6%)	84 (65.6%)	8 (22.9%)	25 (21.7%)	5 (29.4%)	15 (15.3%)	5 (55.6%)	41 (41.0%)
No deliberate	14 (34.1%)	33 (23.4%)	27 (71.1%)	69 (51.5%)	17 (77.3%)	69 (65.7%)	4 (40.0%)	37 (35.6%)
**Deliberate**	27 (65.9%)	108 (76.6%)	11 (28.9%)	65 (48.5%)	5 (22.7%)	36 (34.3%)	6 (60%)	67 (64.4%)
First pregnancy	21 (50.0%)	76 (53.9%)	21 (55.3%)	51 (37.5%)	11 (50.0%)	45 (41.3%)	6 (60.0%)	66 (61.7%)
**Multiparous**	21 (50.0%)	65 (46.1%)	17 (44.7%)	85 (62.5%)	11 (50.0%)	64 (58.7%)	4 (40.0%)	41 (38.3%)
No prenatal question	19 (45.2%)	67 (48.2%)	8 (22.2%)	26 (20.0%)	2 (10.0%)	21 (20.4%)	6 (60.0%)	50 (51.5%)
**Prenatal question**	23 (54.8%)	72 (51.8%)	28 (77.8%)	104 (80.0%)	18 (90.0%)	82 (79.6%)	4 (40.0%)	47 (48.5%)
No posnatal question	15 (36.6%)	55 (41.4%)	4 (11.8%)	23 (20.7%)	2 (11.1%)	15 (16.1%)	4 (66.7%)	50 (50.0%)
**Prenatal question**	26 (63.4%)	78 (58.6%)	30 (88.2%)	88 (79.3%)	16 (88.9%)	78 (83.9%)	2 (33.3%)	50 (50.0%)

Supll. Table S4. Prevalence of health characteristics based on race.

	White		Hispanic		Black		Asian	
	Help	No help	Help	No help	Help	No help	Help	No help
Good perception of health	39 (92.9%)	138 (98.6%)	28 (73.7%)	116 (85.3%)	19 (86.4%)	100 (91.7%)	9 (90.0%)	98 (91.6%)
**Poor perception of health**	3 (7.1%)	2 (1.4%)	10 (26.3%)	20 (14.7%)	3 (13.6%)	9 (8.3%)	1 (10.0%)	9 (8.4%)
No Medicaid	36 (85.7%)	101 (77.7%)	14 (38.9%)	53 (42.7%)	11 (52.4%)	53 (51.5%)	8 (80.0%)	58 (58.6%)
**Medicaid**	6 (14.3%)	29 (22.3%)	22 (61.1%)	71 (57.3%)	10 (47.6%)	50 (48.5%)	2 (20.0%)	41 (41.4%)
NO WIC	39 (92.9%)	116 (83.5%)	13 (34.2%)	51 (38.1%)	7 (31.8%)	42 (38.5%)	9 (90.0%)	56 (53.3%)
**WIC**	3 (7.1%)	23 (16.5%)	25 (65.8%)	83 (61.9%)	15 (68.2%)	67 (61.5%)	1 (10.0%)	49 (46.7%)
No postpartum checkup	1 (2.4%)	5 (3.6%)	4 (10.5%)	24 (17.8%)	4 (18.2%)	16 (14.8%)	4 (40.0%)	6 (5.7%)
**Postpartum checkup**	41 (97.6%)	135 (96.4%)	34 (89.5%)	111 (82.2%)	18 (81.8%)	92 (85.2%)	6 (60.0%)	100 (94.3%)
No depression	28 (70.0%)	125 (90.6%)	23 (62.2%)	113 (84.3%)	18 (81.8%)	93 (88.6%)	8 (80.0%)	100 (95.2%)
**Depression**	12 (30.0%)	13 (9.4%)	14 (37.8%)	21 (15.7%)	4 (18.2%)	12 (11.4%)	2 (20.0%)	5 (4.8%)
No anxiety	18 (43.9%)	111 (80.4%)	20 (52.6%)	106 (79.1%)	17 (77.3%)	94 (88.7%)	7 (70.0%)	94 (89.5%)
**Anxiety**	23 (56.1%)	27 (19.6%)	18 (47.4%)	28 (20.9%)	5 (22.7%)	12 (11.3%)	3 (30.0%)	11 (10.5%)
No visit for dep./ anxiety	26 (65.0%)	103 (92.8%)	20 (69.0%)	65 (95.6%)	16 (94.1%)	70 (94.6%)	4 (66.7%)	74 (100.0%)
**Visit for dep./ anxiety**	14 (35.0%)	8 (7.2%)	9 (31.0%)	3 (4.4%)	1 (5.9%)	4 (5.4%)	2 (33.3%)	0 (0.0%)
No visit for disease	30 (75.0%)	100 (90.1%)	24 (82.8%)	68 (100.0%)	14 (82.4%)	72 (97.3%)	6 (100.0%)	66 (89.2%)
**Visit for disease**	10 (25.0%)	11 (9.9%)	5 (17.2%)	0 (0.0%)	3 (17.6%)	2 (2.7%)	0 (0.0%)	8 (10.8%)
